# Light Absorption and Scattering Properties of Ag@TiO_2_ Nanosphere Dimer for Photocatalytic Water Purification

**DOI:** 10.3390/nano15211618

**Published:** 2025-10-23

**Authors:** Bojun Pu, Paerhatijiang Tuersun, Shuyuan Li, Guoming He, Fengyi Dou, Shuqi Lv

**Affiliations:** Xinjiang Key Laboratory of Luminescence Minerals and Optical Functional Materials, School of Physics and Electronic Engineering, Xinjiang Normal University, Urumqi 830054, China; 107622023210497@stu.xjnu.edu.cn (B.P.); 107622024210501@stu.xjnu.edu.cn (G.H.); 107622024210502@stu.xjnu.edu.cn (F.D.); 107622023210484@stu.xjnu.edu.cn (S.L.)

**Keywords:** core–shell nanosphere dimer, photocatalytic water purification, light absorption and scattering, finite element method, photothermal

## Abstract

Finding high-performance and low-cost materials is essential for high-quality photocatalytic water purification to expand the spectral response and improve light utilization. In this paper, we used relatively inexpensive materials such as Ag and TiO_2_. The influence of particle spacing, core radius, shell thickness, environmental refractive index, and incident light direction angle on the light absorption and scattering properties, local electric field enhancement, and photothermal effect of the Ag@TiO_2_ core–shell nanosphere dimer is investigated by using the finite element method and the finite difference time domain. The formation mechanism of multipole resonance mode of the dimer is revealed by means of the multipole decomposition theory and the internal current distribution of the particles. The results show that light absorption and scattering of the dimer can be tuned within the visible light range by changing the particle spacing, core radius, and shell thickness. With the azimuth angle of incident light increases, the longitudinal local surface plasmon resonance (L-LSPR) mode will transform into the transverse local surface plasmon resonance (T-LSPR) mode, and the L-LSPR mode makes the dimer have better local electric field enhancement. Strong light absorption can easily cause a sharp increase in the temperature around the dimer, accelerating the rate of catalytic oxidation reactions and the elimination of bacteria and viruses in water. Strong light scattering causes a significant enhancement of the electric field between the particles, making the generation of hydroxyl and other active oxides more efficient and convenient. This work establishes a theoretical basis for designing efficient water purification photocatalysts.

## 1. Introduction

Water is the most precious and vital resource in the world. Countless freshwater resources worldwide have been polluted due to industrial activities and human negligence. Water pollution and shortage have become one of the most serious problems in the world today, and it is urgent for people to purify and treat water resources [1,2,3,4]. Wastewater purification refers to the process of removing unwanted chemical substances, suspended solids, and biological contaminants from water systems, in order to produce clean and safe water for human consumption and other uses. The traditional method for treating wastewater involves using filtration and adsorption–precipitation to remove the larger suspended solids in water and then employing chlorination and ultraviolet irradiation to oxidize and decompose microorganisms and other toxic and harmful impurities. Chlorination utilizes the strong oxidizing property of chlorine gas to oxidize and decompose pollutants in water and is widely used in the sewage treatment systems of various cities. Although effective for pollutant degradation, long-term chlorination can lead to pathogenic bacteria developing resistance [5,6,7] and excessive residual chlorine, posing serious threats to human health [8,9]. Compared with chlorination, ultraviolet light (UV) water purification is cleaner and safer. The wavelength of UV is shorter than that of visible light, and thus it has higher energy. When the UV energy is incident upon water, the pollutants in the water will absorb the UV energy and break the chemical bonds, thereby achieving the effect of decomposing the pollutants. However, its limitations are also obvious. UV water purification not only requires the use of expensive artificial light sources but also has a significant energy loss when directly irradiating the pollutants in water and insufficient utilization. Therefore, the addition of catalysts in water to promote energy utilization has emerged as a solution [10,11].

TiO_2_ is widely used in environmental purification due to its strong antioxidant capacity, high stability, non-toxicity, and low cost [12,13]. TiO_2_ added to water can utilize the energy from UV radiation exceeding its band gap (~3.2 eV). This energy absorption promotes electrons from the valence band to the conduction band (intrinsic absorption), generating free electrons (e^−^) in the conduction band and leaving behind holes (h^+^) in the valence band. The h^+^ has a strong attraction for the bonding electrons in water and pollutants (this attraction becomes even stronger under a strong electric field), which can cause the valence bonds to break, achieving the purpose of oxidation and decomposition. Under the catalytic oxidation reaction caused by the combined action of strong electric field and TiO_2_, the water undergoes catalytic oxidation, and the hydroxyl radicals are produced. Similarly, dissolved oxygen in water and oxygen produced by electrolysis of water under weak electric field on the TiO_2_ surface will gain electrons and generate peroxide ions. Both of them have strong oxidizing ability and are helpful for efficient decomposition of pollutants [14,15,16]. Although TiO_2_ can be used as a catalyst to make up for the shortcoming of energy utilization, it is only limited to the ultraviolet light band. Therefore, it is necessary to expand the spectral response range of photocatalytic materials in the water purification process, as it will effectively enhance their efficiency in degrading pollutants.

Tada et al. [16,17] synthesized dye sensitizers by incorporating Au nanoparticles into the TiO_2_ surface, which can broaden the spectral range. The reason is that the Au nanoparticles in the dye absorb photons to generate localized surface plasmon resonance (LSPR), causing an uneven electron distribution. The electrons occupying the highest molecular orbitals first transfer to the lowest unoccupied molecular orbitals, and then to the conduction band of TiO_2_ [18]. The Au nanoparticles, lacking electrons, act as h^+^, and the h^+^ can oxidize and decompose pollutants, thereby achieving the effect of decontamination. When UV light is incident on water, TiO_2_ plays a major role, while when visible light is incident on water, Au nanoparticles play a major role. The two complement each other to achieve the purpose of water purification. Au nanoparticles are not the most effective noble metal for LSPR and are expensive. In contrast, Ag nanoparticles exhibit superior LSPR properties [19] and are significantly lower in cost than Au. Therefore, Ag nanoparticles have great commercial potential for making water purifiers. However, the disadvantages of Ag nanoparticles, such as instability, easy oxidation, and toxicity, limit their application. Hong et al. [20] encapsulated the outer layer of Ag nanoparticles with TiO_2_ to form Ag@TiO_2_ core–shell nanoparticles. They characterized the structure of these nanoparticles using scanning electron microscopy, transmission electron microscopy, and X-ray diffraction. Spectral observations revealed a redshift in the absorption peak to the visible light range, indicating that their light-responsive range expanded to the visible light region. They used it as a photocatalyst and successfully achieved the conversion of CO_2_ to CH_4_ under a CO_2_ atmosphere by irradiation with a solar simulator (AM1.5). However, they did not deeply investigate the theoretical mechanism of action. In the previous work of our group [21], the study on the light absorption properties of nanorods with Ag as the core and TiO_2_ as the shell were theoretically calculated and optimized. By adjusting the shell thickness, particle aspect ratio, and incident light direction angle, the light scattering of Ag@TiO_2_ throughout the near-infrared biological window range was regulated. Although the core–shell structure compensates for the deficiencies and shortcomings of Ag, since the Ag nanoparticles are encapsulated inside the particles, the h^+^-generated due to the lack of electrons cannot be exposed, which leads to a decrease in its oxidizing property.

To address this issue, compared with the previous articles [21] that only reported the single-particle photothermal effect, this study delved deeper into the photothermal absorption and scattering properties when there is interaction coupling between the particles. Two TiO_2_-coated spherical Ag nanospheres were used, and the defect was compensated by forming Ag@TiO_2_ core–shell nanospheres dimer through bringing them close to each other. The reasons are as follows: (1) The strong scattering property of the dimer towards light causes a significantly enhanced local electric field around it. Under the influence of a strong electric field, water molecules are prone to undergo electrolysis and generate hydroxyl radicals, and the stronger the electric field, the easier it is to generate [22]; (2) After the silver nanospheres undergo LSPR, they produce an uneven distribution of free electrons through the Schottky junction formed between Ag and TiO_2_, which are then transferred to the surface by the by-products of water electrolysis (oxygen) and dissolved oxygen in water, forming peroxide ions [23]; (3) The absorption property of the dimer towards light energy causes a sharp increase in temperature nearby. Under the dual effects of various oxidants generated by the strong electric field catalysis and high temperature, the oxidation and decomposition of pollutants by the dimer becomes reasonable. Due to its low cost and high efficiency, Ag@TiO_2_ shell nanospheres have become an ideal choice for applications such as pollution control and antibacterial coatings.

This paper provides a detailed investigation on the light absorption and scattering properties of Ag@TiO_2_ core–shell nanosphere dimer. We analyze the influence of internal factors (particle spacing, core radius, shell thickness) and external factors (environmental refractive index, incident light direction angle) on the resonance peak positions, electric field enhancement, multipole resonance peak current density distribution, and temperature field distribution of light absorption and scattering. We also present the universal laws governing the changes in the absorption and scattering properties of Ag@TiO_2_ core–shell nanosphere dimer and the temperature field distribution in response to various influencing factors.

## 2. Materials and Methods

The light absorption and scattering properties of nanosphere dimer can be simulated using analytic method and various numerical methods such as Mie theory method, discrete dipole approximation (DDA), boundary element method (BEM), finite difference time domain (FDTD), and finite element method (FEM) [24,25,26]. In this paper, the optical and thermal components of the Ag@TiO_2_ shell nanosphere dimer are mainly calculated by using the finite element method combined with the refractive index size correction model for numerical simulation, while the decomposition of the multipole resonance peaks is carried out by using the finite difference time domain method combined with the multipole decomposition theory to decompose the multipole resonance peaks of the dimer.

### 2.1. Simulation Model

**Figure 1:**
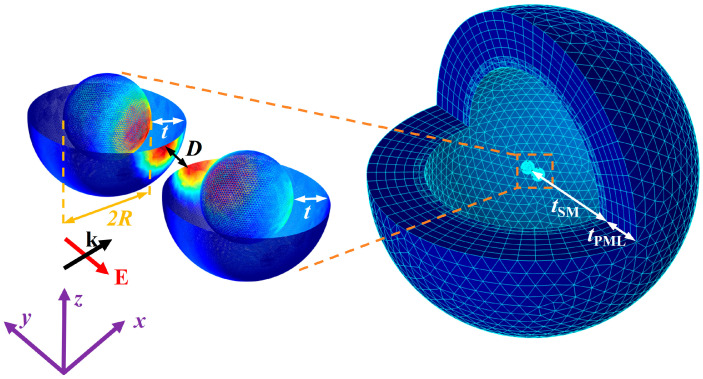
Geometric shape and simulation area of the Ag@TiO_2_ core–shell nanosphere dimer. *R* is the radius of the core, *D* is the particle spacing, *t* is the thickness of the shell, the light incident direction is along the positive *x*-axis, the electric field oscillation direction is along the *y*-axis, *t*_SM_ is the thickness of the surrounding environment layer, and *t*_PML_ is the thickness of the perfect matching layer.

In Figure 1, we present the simulation model of Ag@TiO_2_ core–shell nanosphere dimer and the computational area. The Ag@TiO_2_ core–shell nanosphere dimer consists of two Ag nanosphere cores and a uniform TiO_2_ nanoshell. The geometric structure can be described by the following three parameters: the radius *R* of core, the thickness *t* of shell, and the particle spacing *D*. In the simulation, the incident light wavelength is *λ*, the propagation direction is along the positive *x*-axis, the electric field oscillation direction is along the *y*-axis, and the line connecting the centers of the two particles defines the axis direction of the dimer. The surrounding medium (SM) outside the particle is a spherical region with its center at the midpoint between the two particles, and a radius of (*D*/2 + 2*R* + 2*t* + *λ*/2). The thickness of this SM layer is *t*_SM_, and the SM layer defines the area for calculating the light absorption and scattering parameters. Outside the SM layer, take a spherical region with a thickness of *t*_PML_ equal to *λ*/4 as the perfectly matched layer (PML). PML is an artificially set absorbing boundary condition. In the simulation, it truncates the calculation area, equivalent to a physical problem with an open boundary. When light hits the PML layer, the irradiance is reduced to zero and the light is completely absorbed by it. This is consistent with the traditional view that when light propagates to infinity, the irradiance is zero. The outermost boundary of the PML layer is set as a scattering boundary condition to ensure that the light is completely absorbed by the PML.

### 2.2. Calculation of Absorption and Scattering Properties

The light absorption and scattering capabilities of nanoparticle dimers can be quantitatively described by the absorption cross-section (*C*_abs_) and the scattering cross-section (*C*_sca_). The absorption cross-section is defined as the ratio of the absorbed light energy (*W*_a_) of the dimer to the incident irradiance (*I*_i_), while the scattering cross-section is defined as the ratio of the scattered light energy (*W*_s_) of the dimer to the incident irradiance (*I*_i_). They can be expressed as follows:

(1) $$C_{\mathrm{abs}}=\frac{W_{a}}{I_{i}},$$

(2) $$C_{\mathrm{sca}}=\frac{W_{s}}{I_{i}},$$

where the incident irradiance $I_{i}=(n_{m}E_{0}^{2})/(2Z_{0})$, *n*_m_ is the refractive index of the SM, *E*_0_ is the intensity of the background electric field, and Z_0_ is the impedance of free space.

The number of substances contained in different-sized nanoparticle dimers varies, resulting in different abilities to absorb and scatter light. To fairly compare the light absorption and scattering capabilities of nanoparticle dimers, in this paper, the absorption cross-section and scattering cross-section are evenly distributed over the unit volume. The absorption cross-section and scattering cross-section on the unit volume are referred to as the volume absorption coefficient *A*_abs_ and the volume scattering coefficient *A*_sca_, and their expressions are as follows:

(3) $$A_{\mathrm{abs}}=\frac{C_{\mathrm{abs}}}{V},$$

(4) $$A_{\mathrm{sca}}=\frac{C_{\mathrm{sca}}}{V},$$

where *C*_abs_ and *C*_sca_ represent the absorption and scattering cross-sections of the entire dimer, while V represents the volume of the entire dimer.

When calculating the *A*_abs_ and *A*_sca_ of nanoparticles, the refractive index of the nanoparticles needs to be obtained. When the nanoparticles are metallic, their refractive index is not only a function of the incident light frequency, but also a function of the particle size. For particle sizes smaller than the average free path of the free electrons in the material, the numerous free electrons abundant in the metallic material and their collisions with the surface will significantly enhance, and thus the surface scattering of free electrons must be taken into account [27]. At this time, the expression of the material refractive index of metallic nanoparticles as a function of particle size is [28]:

(5) $$n_{\mathrm{nano}}(\omega ,L_{\mathrm{eff}})=\sqrt{n_{\mathrm{bulk}}^{2}(\omega )+\frac{\omega_{p}^{2}}{\omega^{2}+i\omega v_{f}/l_{\infty }}-\frac{\omega_{p}^{2}}{\omega^{2}+i\omega (v_{f}/l_{\infty }+Av_{f}/L_{\mathrm{eff}})}},$$

where *ω* is the incident light frequency, *n*_bulk_ is experimental data on the refractive index of bulk materials, *ω*_p_ is the plasma frequency, *v*_f_ is called Fermi velocity, *l*_∞_ is the average free path of free electrons, and *A* is a dimensionless parameter (it equals to 1 in this paper). The effective average free path of free electrons *L*_eff_ is usually taken as *R*. For Ag, *n*_bulk_ is derived based on the experimental data published by Johnson and Christy [29]. The plasma frequency ℏ*ω*_p_ is 9.01 eV [30], the Fermi velocity *v*_f_ is 1.39 × 10^6^ m/s [31], and the average free path of free electrons *l*_∞_ is 52 nm [31]. The refractive index of TiO_2_ is acquired with the experimental data published by Sharma et al. [32].

### 2.3. Multipole Decomposition of Scattered Spectra

The total electric field $E(r)\;=\;E_{0}+E_{s}$ outside the particle can be obtained by the finite element method, where $E_{0}$ is the background electric field intensity, $E_{s}$ is the scattering electric field intensity, and r =(x,y,z) is the spatial position vector. When a plane wave is incident on a nanoparticle dimer, the current density distribution induced and excited is expressed as

(6) $$J(r)=-i\omega \varepsilon_{0}(n^{2}-1)E(r),$$

where *ω* is the angular frequency of light, ε_0_ is the vacuum dielectric constant, and *n* is the complex refractive index of the dimer particles. The electric dipole (**p**), magnetic dipole (**m**), electric quadrupole (**Q**^e^) and magnetic quadrupole (**Q**^m^) can be expressed as [33]:

(7) $$\begin{array}{l} p_{\alpha }=-\frac{1}{i\omega }\left[\int J_{\alpha }j_{0}(\mathrm{kr})d^{3}r+\frac{k^{2}}{2}\int \{3(r\cdot J)r_{\alpha }-r^{2}J_{\alpha }\}\frac{j_{2}(\mathrm{kr})}{{\left(kr\right)}^{2}}d^{3}r\right],\\ m_{\alpha }=\frac{3}{2}\int {\left(r\times J\right)}_{\alpha }\frac{j_{1}(\mathrm{kr})}{kr}d^{3}r,\\ Q_{\alpha \beta }^{e}=-\frac{3}{i\omega }\left[\begin{array}{l} \int \{3(r_{\beta }J_{\alpha }+r_{\alpha }J_{\beta })-2(r\cdot J)\delta_{\alpha \beta }\}\frac{j_{1}(\mathrm{kr})}{kr}d^{3}r\\ +2k^{2}\int \{5r_{\alpha }r_{\beta }(r\cdot J)-r^{2}(r_{\alpha }J_{\beta }+r_{\beta }J_{\alpha })-r^{2}(r\cdot J)\delta_{\alpha \beta }\}\frac{j_{3}(\mathrm{kr})}{{\left(kr\right)}^{3}}d^{3}r \end{array}\right],\\ Q_{\alpha \beta }^{e}=15\int \{r_{\alpha }{\left(r\times J\right)}_{\beta }+r_{\beta }{\left(r\times J\right)}_{\alpha }\}\frac{j_{2}(\mathrm{kr})}{{\left(kr\right)}^{2}}d^{3}r, \end{array}$$

where α,β=x,y,z, *k* represents the wave number, and $j_{n}(\rho )$ is the spherical Bessel function, which is defined as $j_{n}(\rho )=\sqrt{\pi /2\rho }J_{n+1/2}(\rho )$. $J_{n}(\rho )$ is the first kind of cylindrical Bessel function. Using the aforementioned multipole moments, the total scattering cross-section can be expressed as [34]

(8) $$C_{\mathrm{sca}}^{\mathrm{total}}=\frac{k^{4}}{6\pi \varepsilon_{0}^{2}|E_{0}|}\left[\sum \left(|p{|}^{2}+{\left|\frac{m}{c}\right|}^{2}\right)+\frac{1}{120}\sum \left({\left|Q^{e}\right|}^{2}+{\left|\frac{kQ^{m}}{c}\right|}^{2}\right)+...\right].$$

From Equation (8), it can be seen that the total scattering cross-section is simply the sum of the partial scattering cross-sections from different multipole ($C_{\mathrm{sca}}^{p},C_{\mathrm{sca}}^{m},C_{\mathrm{sca}}^{Q^{e}},C_{\mathrm{sca}}^{Q^{m}}$) components.

### 2.4. Heat Transfer Calculation

To investigate the thermal behavior of the Ag@TiO_2_ core–shell nanosphere dimer and its impact on the surrounding environment, we employ the time-dependent (transient heat) equation [35]:

(9) $$C_{p}\rho \frac{\partial T}{\partial t}+\nabla \cdot (-k\nabla T)=Q,$$

where *C*_p_ represents the isobaric heat capacity, *ρ* denotes the density, *k* is the thermal conductivity, and *Q* is the heating power per unit volume. The relevant information on the materials used in this article is provided in Table 1.

## 3. Results and Discussion

To ensure precision and dependability of finite element modeling for dimer, this paper first validates the simulation results. Then, the FEM is used to quantitatively analyze the influence of the size parameters (particle spacing, core radius, shell thickness) of Ag@TiO_2_ core–shell nanosphere dimer and external factors (SM refractive index, incident light direction) on the resonance peak positions of light absorption and scattering as well as the electric field enhancement effect. In addition, the typical scattering section of the dimer with multiple resonance peaks is decomposed into multipole components, and the current distribution inside the particles is given, further revealing the formation mechanism of multiple resonance peaks. Finally, the thermal process of the Joule Heat generated by the electrons inside the dimer radiating to the surrounding environment is analyzed.

### 3.1. Numerical Verification of FEM

**Figure 2:**
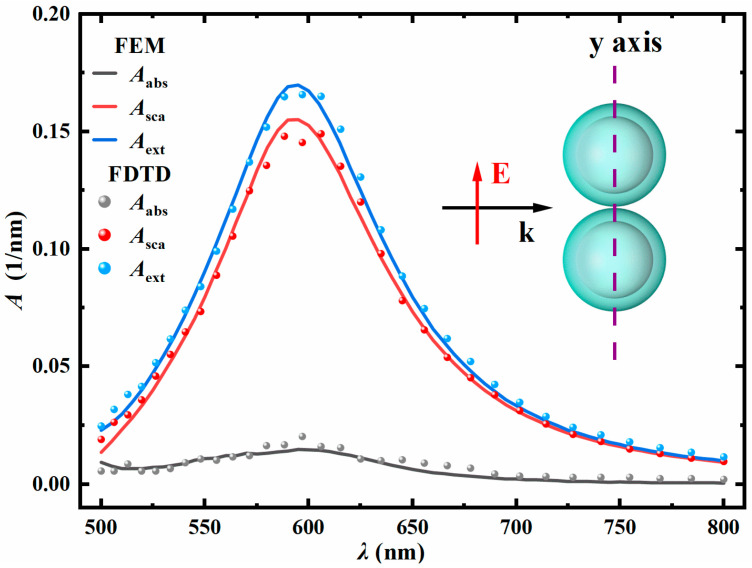
The variations in the volume absorption coefficient (*A*_abs_), volume scattering coefficient (*A*_sca_), and volume extinction coefficient (*A*_ext_) of the dimer with respect to the incident light wavelength *λ*. *R* = 30 nm, *t* = 10 nm, *D* = 1 nm, *n*_m_ = 1.33, the light incident direction along the positive *x*-axis, and the electric field polarization direction along the *y*-axis.

In this paper, the optical properties of the same dimer (with exactly the same parameters) were numerically calculated using both FEM and FDTD methods. The accuracy and reliability of FEM were verified by comparing the calculation results. As shown in Figure 2, the volume absorption coefficient (*A*_abs_), volume scattering coefficient (*A*_sca_), and volume extinction coefficient (*A*_ext_) of the Ag@TiO_2_ core–shell nanosphere dimer calculated by the two methods are presented as a function of the incident light wavelength. The inner core radius *R* is 30 nm, the shell thickness *t* is 10 nm, the particle spacing *D* is 1 nm, and the refractive index of the surrounding environment *n*_m_ is 1.33. The incident light direction is along the positive *x*-axis, and the electric field polarization direction is along the *y*-axis. The results show that the calculation results of FEM are in good agreement with the simulation results of FDTD, verifying precision and dependability of finite element modeling.

### 3.2. The Influence of Internal Factors Within the Dimer

This section quantitatively analyzed the effects of size parameters such as particle spacing, core radius, and shell thickness on the spectra, scattering spectra, and electric field enhancement of Ag@TiO_2_ core–shell nanosphere dimer.

#### 3.2.1. The Influence of Particle Spacing

**Figure 3:**
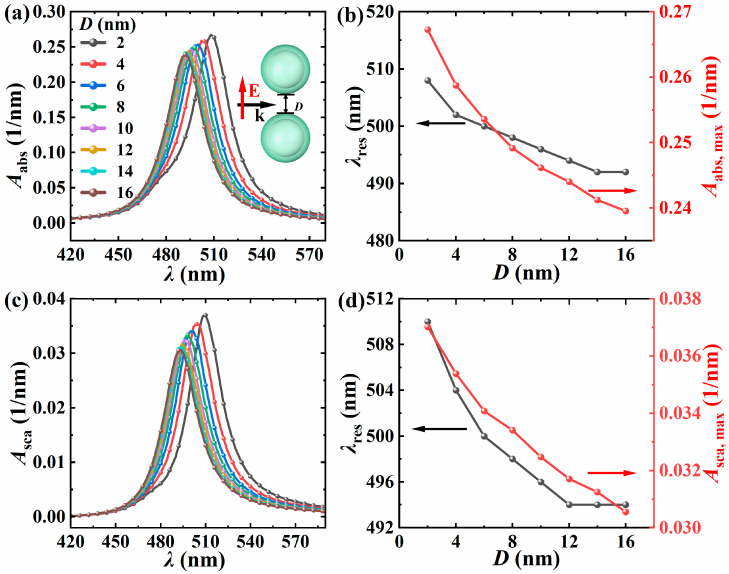
The variations of *A*_abs_ (**a**) and *A*_sca_ (**c**) of different *D* with the *λ*. The resonance wavelength *λ*_res_ of the dimer, the peak value of *A*_abs_ (**b**), and the peak value of *A*_sca_ (**d**) change with the *D*. The *R* is 10 nm, the *t* is 5 nm, the *n*_m_ is 1.33, the light incident direction is along the positive *x*-axis, and the electric field polarization direction is along the *y*-axis.

**Figure 4:**
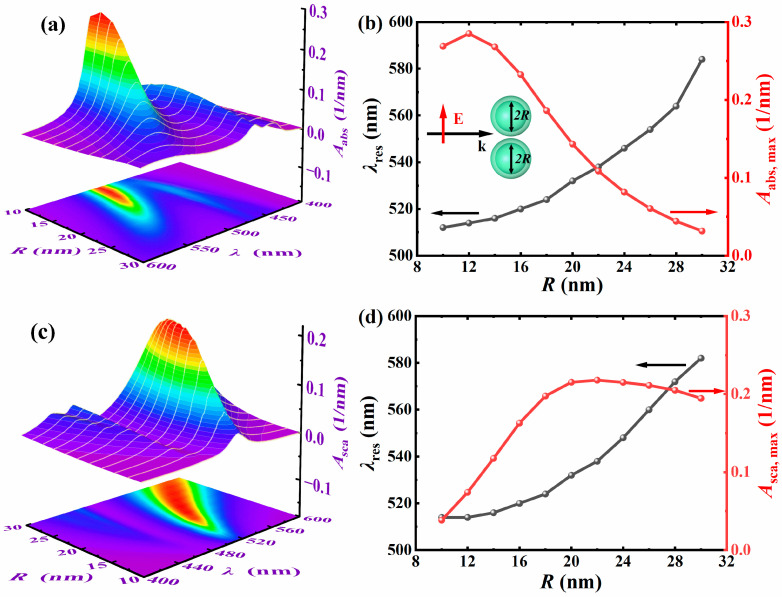
The variations of *A*_abs_ (**a**) and *A*_sca_ (**c**) of different *R* with the *λ*. The maximum resonance wavelength *λ*_res_ of the dimer and the peaks of *A*_abs_ (**b**), and the peak of *A*_sca_ (**d**) vary with the *R*. The *t* is 5 nm, the *D* is 1 nm, the *n*_m_ is 1.33, the incident light direction is along the positive *x*-axis, and the electric field polarization direction is along the *y*-axis.

Figure 3 shows the absorption spectrum and scattering spectrum of the dimer, with the core radius *R* = 10 nm, shell thickness *t* = 5 nm, surrounding environment refractive index *n*_m_ = 1.33, light incident direction along the positive *x*-axis, electric field polarization direction along the *y*-axis, particle spacing *D* increasing from 2 nm to 16 nm (step size of 2 nm), the variation in resonance wavelength and peak with the dimer spacing. Figure 3a,c show prominent dipole resonance peaks in the visible light band, an effect attributed to the particle size being much smaller than the incident wavelength. From Figure 3b,d, it is evident that the resonance wavelength shifts towards the red as the particle spacing decreases, and the redshift speed gradually increases. This is because when the dimer spacing decreases, the particles undergo near-field coupling, and the coupling effect causes the electron movement frequency to decrease, resulting in a redshift in the resonance wavelength. In addition, the core and shell plasmons of a single particle hybridize and split into bonding and antibonding modes. The antibonding mode has higher energy (dark mode), while the symmetric mode has lower energy and matches a longer wavelength of light. The resonance wavelength shifts towards the red by [37]. When the particle spacing decreases to around 2 nm, spectral lines begin to show slight asymmetry, and this becomes more obvious when the particle spacing decreases to 1 nm (see Figure 4a). This asymmetry is caused by the change in the plasmon coupling mode between particles. During the process of decreasing the spacing, due to the enhanced near-field coupling between particles, the accumulation of charges between particles leads to an increase in charge density, an increase in local electric field, and electrons need more light energy to overcome the obstruction of the local electric field and do work, directly increasing the absorption and scattering power, thereby increasing the peak values of the volume absorption and volume scattering coefficients. Therefore, the smaller the particle spacing, the stronger the absorption and scattering ability of the dimer.

#### 3.2.2. The Impact of Kernel Radius

Subsequently, the influence of the core radius on the absorption and scattering spectra of Ag@TiO_2_ core–shell nanosphere dimer is analyzed. The thickness of the shell structure *t* is 5 nm, the particle spacing *D* = 1 nm, the surrounding refractive index *n*_m_ = 1.33, the light incident direction is along the positive *x*-axis, and the electric field polarization direction is along the *y*-axis. Figure 4a,c, respectively, show the absorption spectra and scattering spectra when the core radius increases from 10 nm to 30 nm (step size of 2 nm). Figure 4b,d show the variation in the maximum resonant wavelength and the corresponding peak of the dimer with the core radius. The results indicated that as the core radius increased, the resonant wavelength redshifts of absorption spectra and scattering spectra, and the redshift speed gradually accelerated. When the core radius is 10 nm, there is only one electric dipole resonant peak; when the core radius becomes 30 nm, multiple electric dipoles, electric quadrupole, and other resonant peaks are generated. With the increase in the core radius, the number of resonant peaks gradually increases, and if the core radius continues to increase, the resonance mode would become more complex. Interestingly, both the absorption and scattering resonant peaks show a trend of increasing first and then decreasing. When the particle size is small, absorption dominates; however, as the size increases, scattering gradually becomes dominant. This is because the increase in the core radius caused the originally degenerate dipole and quadrupole modes to split. When the ratio of the core radius to the shell radius (*R*_core_/*R*_shell_) increases, the interaction between the bonding mode and the antibonding mode is enhanced, thereby increasing the number of resonant modes, and the new resonant peaks split from the original resonant peaks shows a trend of increasing first and then decreasing.

**Figure 5:**
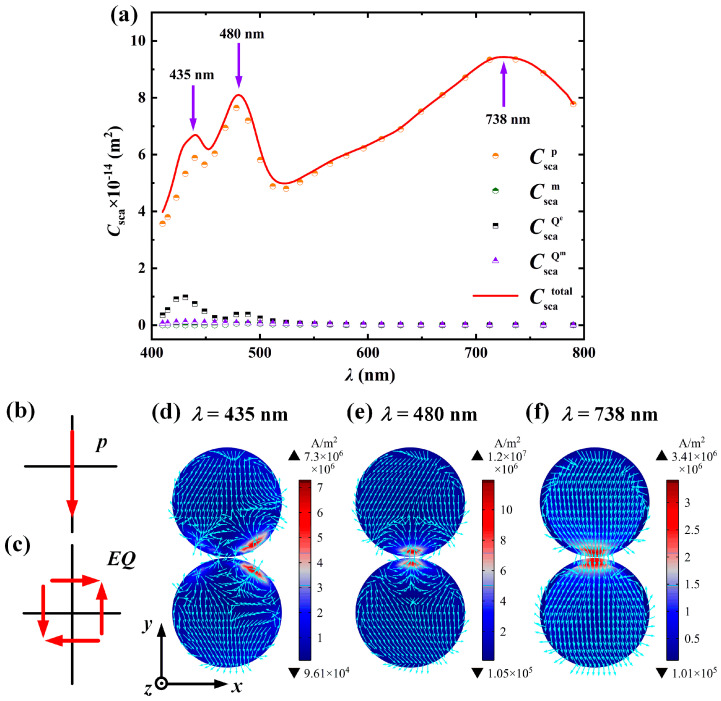
Scattering spectra multipole decomposition and current density distribution of Ag@TiO_2_ core–shell nanosphere dimer. (**a**) Multipole decomposition diagram of the scattering cross-section *C*_sca_ as a function of incident wavelength *λ*, where $C_{\mathrm{sca}}^{p}$, $C_{\mathrm{sca}}^{m}$, $C_{\mathrm{sca}}^{Q^{e}}$, and $C_{\mathrm{sca}}^{Q^{m}}$ represent the scattering cross-section changes caused by electric dipole resonance, magnetic dipole resonance, and electric quadrupole resonance, respectively; (**b**) Schematic diagram of the current density direction within a single nanosphere during electric dipole resonance; (**c**) Schematic diagram of the current density direction during electric quadrupole resonance; (**d**) Current density distribution diagrams at wavelengths of 435 nm, (**e**) 480 nm, and (**f**) 738 nm. The colors in the figures represent current intensity, and the arrows indicate current direction. The *R* is 50 nm, the *t* is 5 nm, the *D* is 1 nm, the *n*_m_ is 1.33, the incident light direction is along the positive *x*-axis, and the electric field polarization direction is along the *y*-axis.

To further illustrate the causes of each peak, a specific-sized nanosphere dimer is selected for multipole decomposition. Since the small particle only excites the electric dipole resonance mode, while the large particle, due to the retardation effect, cannot uniformly polarize the charges in different regions of its surface, thus exciting higher-order multipole resonance modes (such as electric quadrupole resonance, magnetic quadrupole resonance, etc.), a particle with a slightly larger core radius is chosen to explore the formation mechanism of its multipole resonance peaks. The simulation results are shown in Figure 5. In the simulation, the core radius of a single particle *R* is 50 nm, the shell thickness *t* is 5 nm, the particle spacing *D* is 1 nm, the surrounding medium refractive index *n*_m_ is 1.33, the incident wave direction is along the positive *x*-axis, and the electric field polarization direction is along the *y*-axis. Figure 5a presents the multipole decomposition of the scattering cross-section *C*_sca_ of Ag@TiO_2_ core–shell nanosphere dimers as a function of the incident wavelength *λ*. It can be seen from the figure that the total scattering cross-section has three resonance peaks, with the corresponding wavelengths being 435 nm, 480 nm, and 738 nm. The rightmost resonance peak is entirely excited by electric dipole resonance, while the left two resonance peaks are excited by both electric dipole and electric quadrupole resonances. Figure 5b,c, respectively, show the schematic diagrams of the current direction inside a single nanosphere at electric dipole and electric quadrupole resonances; Figure 5d–f, respectively, present the current density distribution at 435 nm, 480 nm, and 738 nm. It can be seen from Figure 5a,f that the 738 nm resonance peak is completely excited by the electric dipole resonance, that is, the maximum resonance peak in Figure 4 is completely excited by the dipole resonance. From Figure 4b,d, it can be observed that the increase in the core radius leads to a redshift in the electric dipole resonance peak. The reason is that the increase in the Ag core radius will cause the average free path of electron movement to increase, thereby extending the collective oscillation path of the conducting electrons. According to the classical plasma theory, the oscillation frequency of the electron cloud is inversely proportional to its effective movement distance. At the same time, as the core radius increases, the restoring force of the electron oscillation (provided by the polarization field at the core–shell interface) weakens, resulting in a decrease in the resonance frequency. Correspondingly, the wavelength of the pure electric dipole resonance peak redshifts, that is, the resonance frequency decreases, the wavelength becomes longer, and the resonance peak redshifts. It can be found from Figure 5d,e that although both contain quadrupole resonance, the different proportions of resonance modes between particles lead to different current density distributions. In Figure 5e, the electric dipole resonance contributes significantly to the entire resonance peak, and the current density distribution is mainly affected by the electric dipole resonance, which is distributed in the particle gap. However, the reason why the current density distribution in Figure 5d is different from that in the electric quadrupole resonance is that at 435 nm both the electric dipole resonance and the electric quadrupole resonance exist simultaneously. Although the proportion of the electric quadrupole resonance is significantly enhanced, the electric dipole resonance still dominates (the dipole resonance scattering cross-section at 435 nm in Figure 5a is six times higher than the quadrupole resonance scattering cross-section), making it impossible to form the same current distribution as the electric quadrupole resonance, thus causing a lateral shift in the position of the maximum current density distribution.

#### 3.2.3. The Influence of Shell Thickness

**Figure 6:**
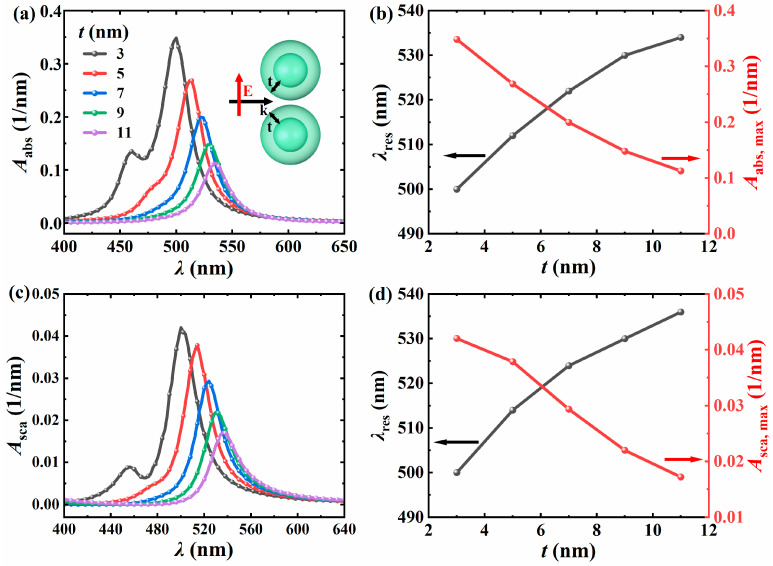
Variation of *A*_abs_ (**a**) and *A*_sca_ (**c**) with incident wavelength *λ* for different *t*. The resonance wavelength *λ*_res_ of the dimer, the peak value of *A*_abs_ (**b**), and the peak value of *A*_sca_ (**d**) change with the *t*. The *R* = 10 nm, the *D* = 1 nm, the *n*_m_ = 1.33, the light incident direction is along the positive *x*-axis, and the electric field polarization direction is along the *y*-axis.

Subsequently, the influence of the shell thickness on the light absorption and scattering spectra of the Ag@TiO_2_ core–shell nanosphere dimer is analyzed. The inner core radius *R* = 10 nm, the particle spacing *D* = 1 nm, the surrounding environment has a refractive index of *n*_m_ = 1.33, the incident light direction is along the positive *x*-axis, and the electric field polarization direction is along the *y*-axis. Figure 6 shows the light absorption spectra, scattering spectra, as well as the changes in resonance wavelength and peak with the shell thickness when the shell thickness varies from 3 nm to 11 nm (step size of 2 nm). The results indicate that as the shell thickness increases, the resonance wavelength shifts towards the red, and the redshift speed gradually slows down. The reason is similar to that of the inner core radius. When the shell thickness increases, the oscillation path of the electron cloud is extended, the Coulomb restoring force between electrons is weakened, and the electron oscillation frequency within the particles decreases, resulting in an increase in the matching light wavelength. Moreover, as the shell thickness increases, the number of resonance peaks also gradually decreases. The reason for this reduction is that the plasmon resonance is mainly dominated by the Ag nanospheres. When the TiO_2_ layer acting as an auxiliary layer becomes thicker, the distance between the two Ag nanospheres increases, and thus gradually transforms into the influence of the particle spacing on the dimer. The larger the spacing between the Ag nanospheres, the lower their light absorption and scattering performance, which has been confirmed at the point where the particle spacing changes.

#### 3.2.4. The Influence of Size on Electric Field Enhancement

**Figure 7:**
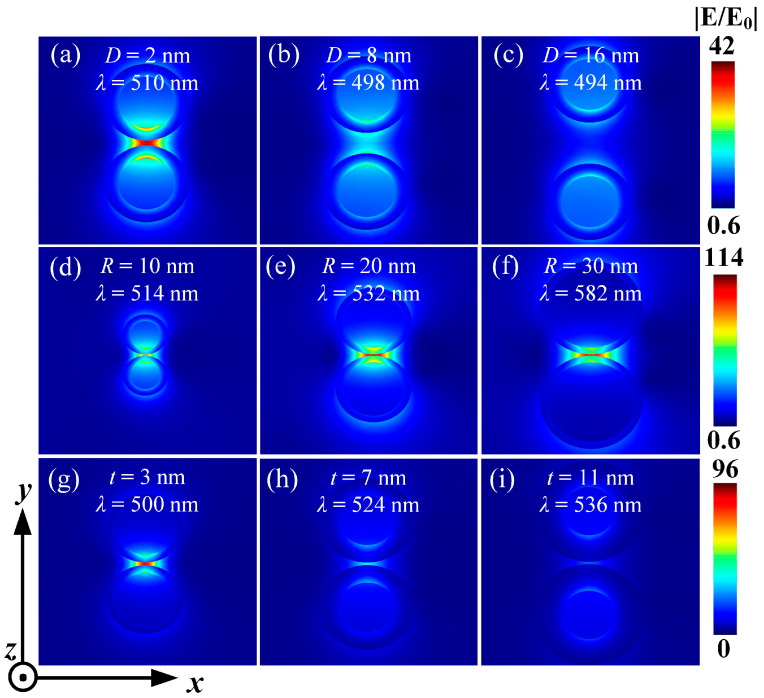
The electric field distribution formed on the *xoy* plane by a plane wave with *y*-axis polarized electric field incident along the positive *x*-axis onto the Ag@TiO_2_ dimer. (**a**–**c**) The electric field distribution changes with the distance between the dimer particles, with the *R* and the *t* fixed at 10 nm and 5 nm, respectively; (**d**–**f**) The electric field distribution changes with the *R* of the particles, with the *t* and the *D* fixed at 5 nm and 1 nm, respectively; (**g**–**i**) The electric field distribution changes with the *t*, with the *R* and *D* fixed at 10 nm and 1 nm, respectively. In all the calculations, the *n*_m_ is 1.33.

Finally, the effects of particle spacing, core radius, and shell thickness on the electric field enhancement of Ag@TiO_2_ core–shell nanosphere dimers were investigated. It was found that the electric field diagrams and spectral diagrams exhibited similar patterns. Figure 7a–c indicate that as the particle spacing gradually increases, the electric field intensity between the particles gradually decreases. When the particle spacing is 2 nm and the resonance wavelength is 510 nm, the electric field enhancement between the particles is the maximum, approximately 42 times the incident field strength. Figure 7d–f show that when the particle radius gradually increases, the electric field intensity between the particles first increases and then decreases. When the core radius is 20 nm and the resonance wavelength is 532 nm, the electric field enhancement between the particles is the maximum, approximately 114 times the incident field strength. Figure 7g–i demonstrate that when the shell thickness gradually increases, the electric field intensity between the particles gradually decreases. When the shell thickness is 3 nm and the resonance wavelength is 500 nm, the electric field enhancement between the particles is the maximum, approximately 96 times the incident field strength. Thus, it can be concluded that when the core radius of the particles is around 20 nm and the particle spacing is smaller, and the shell thickness is thinner, the electric field enhancement effect is relatively better. This suggests that the enhancement is not simply proportional to the Ag volume fraction.

### 3.3. Influence of External Factors on Dimer Structure

This section quantitatively analyzed the effects of the refractive index of the surrounding environment and the incident light direction angle on the absorption spectra, scattering spectra and electric field enhancement of the Ag@TiO_2_ core–shell nanosphere dimer.

#### 3.3.1. The Influence of the Refractive Index of the SM

**Figure 8:**
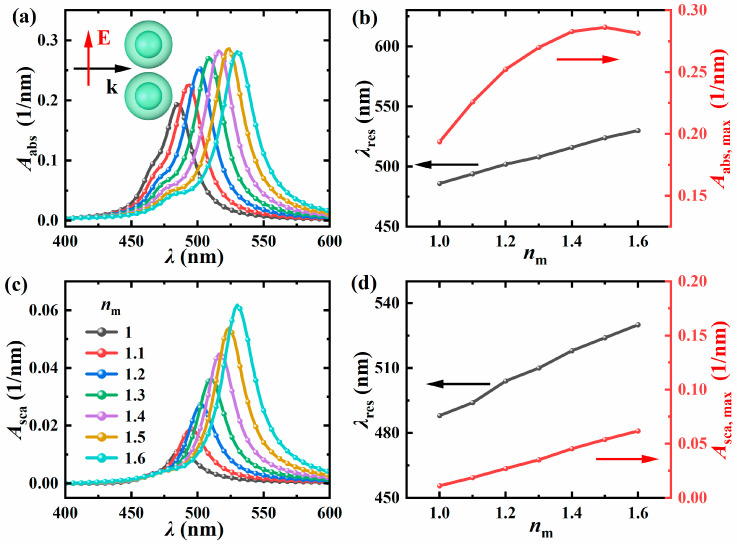
Variation of *A*_abs_ (**a**) and *A*_sca_ (**c**) with incident wavelength *λ* under different *n*_m_. The resonance wavelength *λ*_res_ of the dimer and the peak value of *A*_abs_ (**b**) and the peak value of *A*_sca_ (**d**) change with the *n*_m_. The *R* of the structure is 10 nm, the *t* is 5 nm, the *D* is 1 nm, the light incident direction is along the positive *x*-axis, and the electric field polarization direction is along the *y*-axis.

Since the sewage contains various different substances, the refractive index of the sewage is different from that of pure water to some extent. Therefore, this section discusses the influence of the SM refractive index on the absorption and scattering spectra. Figure 8 shows the absorption spectrum, scattering spectrum, and the changes in resonance wavelength and peak with the SM refractive index when the surrounding refractive index varies from 1 to 1.6 (with a step size of 0.1). The core radius of the structure *R* is 10 nm, the shell thickness *t* is 5 nm, the particle spacing *D* is 1 nm, and the incident light direction is along the positive *x*-axis, and the electric field polarization direction is along the *y*-axis. The results show that the resonance wavelength shifts towards red as the surrounding SM refractive index increases. This is because the plasmon energy of a single nanosphere is affected by the dielectric functions of the core, shell, and surrounding medium together. This viewpoint is based on the paper published by Brandl et al. [37]. For the same core and shell materials, when the SM refractive index increases (the dielectric constant increases), the plasmon energy decreases, resulting in a redshift in the resonance wavelength. Since all the resonance wavelengths of individual particles have shifted towards red, the coupled resonance wavelengths have also shifted towards red.

#### 3.3.2. The Influence of Incident Light Direction Angle

**Figure 9:**
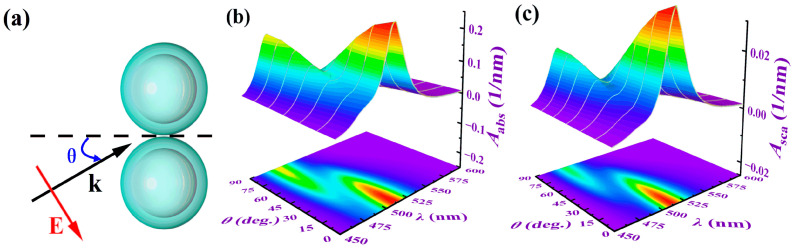
(**a**) Schematic diagram of the incident light direction angle *θ*. Here, the dotted line in the middle of the particle represents the *x*-axis, and the direction angle *θ* is defined as the angle between the wave vector **k** in the *xoy* plane and the positive direction of the *x*-axis. The *A*_abs_ (**b**) and the *A*_sca_ (**c**) of different *θ* vary with the *λ*. Here, the *R* is 10 nm, the *t* is 5 nm, the *D* is 1 nm, and the *n*_m_ is 1.33.

**Figure 10:**
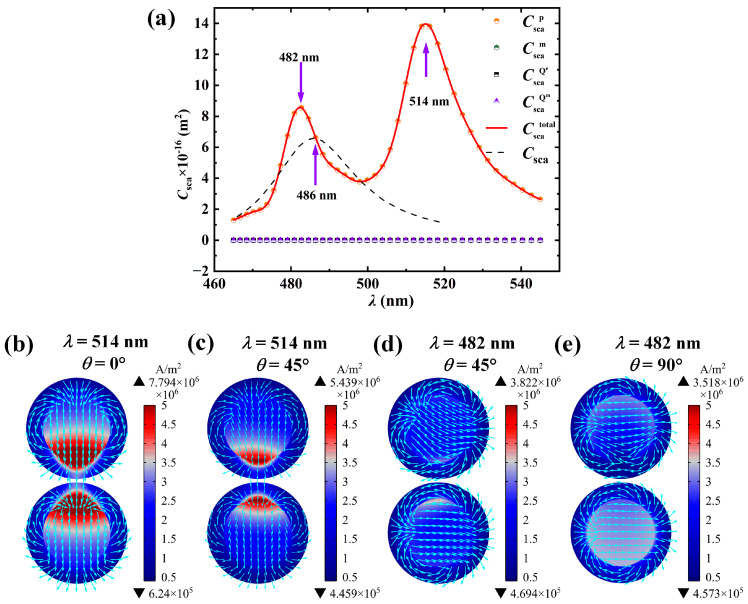
Scattering spectra multipole decomposition and current density distribution of Ag@TiO_2_ core–shell nanosphere dimer. (**a**) Multipole decomposition diagram of the scattering cross-section *C*_sca_ as a function of incident wavelength *λ* when the direction angle is 45°, where $C_{\mathrm{sca}}^{p}$, $C_{\mathrm{sca}}^{m}$, $C_{\mathrm{sca}}^{Q^{e}}$, and $C_{\mathrm{sca}}^{Q^{m}}$ represent the scattering cross-section changes caused by electric dipole resonance, magnetic dipole resonance, electric quadrupole resonance, and magnetic quadrupole resonance, respectively; (**b**,**c**) current density distribution diagrams at 0° and 45° direction angles at 514 nm wavelength; (**d**,**e**) current density distribution diagrams at 45° and 90° direction angles at 482 nm wavelength. In figures (**b**–**e**), colors represent current intensity, and arrows indicate current direction. The *R* is 10 nm, the *t* is 5 nm, the *D* is 1 nm, and the *n*_m_ is 1.33.

We calculated the absorption and scattering spectra of the light as the incident angle changed from 0° to 90° (with a step size of 15°). The results are shown in Figure 9. The core radius of the structure *R* is 10 nm, the shell thickness *t* is 5 nm, the particle spacing *D* is 1 nm, and the surrounding refractive index *n*_m_ is 1.33. The results indicate that for all incident angles except 0° and 90°, there are two resonance peaks in both the absorption and scattering spectra, located at 514 nm and 482 nm, respectively. To further investigate the mechanism of these two resonance peaks, we performed multipole decomposition on the resonance peak of the scattering spectrum at an incident angle of 45° in Figure 9. For comparison, we elongated the particle spacing to 100 nm (so that the coupling effect can be ignored, and the resonance wavelength of the dimer is approximately the same as that of a single nanosphere) and calculated the scattering cross-section at this spacing (the dotted line in the figure). The results are shown in Figure 10a. From Figure 10a, it is found that both of these peaks are dipole resonance peaks. Why do the two dipole resonance peaks occur at different wavelengths?

The study found that there are two modes of oscillation of the current within the dimer, as shown in Figure 10b,e. Figure 10b shows the oscillation mode when the light is incident perpendicularly to the axis of the dimer (incident angle is 0°), which is called the longitudinal localized surface plasmon resonance mode (L-LSPR). Figure 10e shows the oscillation mode when the light is incident perpendicularly to the dimer axis (with a direction angle of 90°). This mode is called the transverse localized surface plasmon resonance mode (T-LSPR). When light obliquely enters the dimer, both modes coexist, but the frequencies of light absorbed by the two modes are different. In Figure 10a, due to the strong coupling effect between the particles, the local electric field of L-LSPR is significantly enhanced. When the free electrons inside the particles move along the dimer axis in the electric field, they are hindered by the local electric field, causing the electron movement frequency to slow down, resulting in a decrease in the frequency of the matching light and a redshift in the resonance wavelength compared to a single nanosphere. In contrast, for T-LSPR, the electrons inside one nanosphere move faster under the electric field generated by the excitation of the other nanosphere, leading to a blueshift in the matching resonance wavelength. This causes the splitting of the electric dipole resonance peak. The coupling effect caused by the particle spacing increases the resonance peak value. However, since the coupling effect between the particles in T-LSPR is relatively weak, the blueshift is smaller and the increase in the peak value is also smaller. This explains the problem that both resonance peaks are dipole resonance peaks but have different resonance wavelengths.

#### 3.3.3. The Influence of SM Refractive Index and Incident Direction Angle on the Enhancement of Electric Field

**Figure 11:**
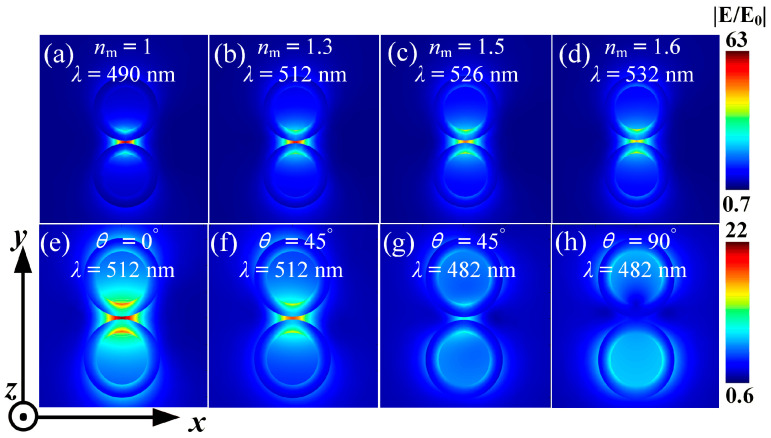
Electric field diagram formed on the *xoy* plane when Ag@TiO_2_ dimer interacts with planar light. (**a**–**d**) Electric field distribution at a direction angle of 0° as the refractive index of the SM changes; (**e**–**h**) Electric field distribution as the *θ* changes when the *n*_m_ is 1.33. In the simulation, the *R* is 10 nm, the *t* is 5 nm, and the *D* is 1 nm.

Figure 11a–d show the variation in the field enhancement of the dimer with the change in the SM refractive index. From the figure, it can be seen that as the SM refractive index increases, the electric field between the particles shows a decreasing trend. The smaller the SM refractive index, the greater the electric field enhancement. This is because when light enters the environment, it causes the SM to polarize. When the SM refractive index is smaller, its polarization rate is smaller, resulting in a smaller polarization intensity of the SM, and thus the generated polarization electric field is smaller. The polarization electric field direction is opposite to the local electric field direction, which weakens the local electric field between the particles. Therefore, when the refractive index is smaller, the field enhancement between the particles is more obvious. In this paper, the maximum electric field enhancement, reaching 63 times, occurs when the *n*_m_ is 1.

As shown in Figure 11e–h, when the incident light direction angle is 0°, the charges are mainly distributed near the center of the dimer. At this time, the L-LSPR is excited, and the maximum electric field enhancement between the particles is 22 times. As the direction angle increases, the charge distribution of the particles rotates counterclockwise [see Figure 10b–e], and thus the L-LSPR gradually becomes a T-LSPR. When the direction angle becomes 45°, the electric field enhancement caused by L-LSPR is very obvious, while the electric field enhancement caused by T-LSPR is relatively weak. After removing the effects of spacing, core radius, shell thickness, and surrounding environment, the remaining electric field enhancement is negligible. When the direction angle becomes 90°, the charge distribution between the particles is less, and the electric field enhancement between the particles gradually weakens, completely manifesting as a T-LSPR. Due to the fact that the incident light is absorbed and scattered by the lower particles first, the electric field distribution inside and near the two particles is not completely consistent. Thus, it can be known that T-LSPR has almost no contribution to the electric field enhancement and can be ignored.

In the above discussion, we have examined the light absorption and scattering properties of the dimer and have derived the variation patterns of its light absorption and scattering characteristics. When the particle spacing and the thickness of the shell are reduced, and the core size is appropriately selected, the strong scattering properties can cause the dimer to generate a high local electric field enhancement, thereby promoting water decomposition to produce hydroxyl radicals and active oxides. Moreover, the high absorption properties can significantly increase the temperature around the particles, and with the dual effect of these properties, the wastewater can achieve the purpose of purification. How high can the temperature rise? We will continue to explore the photothermal properties of the dimer.

### 3.4. Photothermal Effect of Dimer Compounds

**Figure 12:**
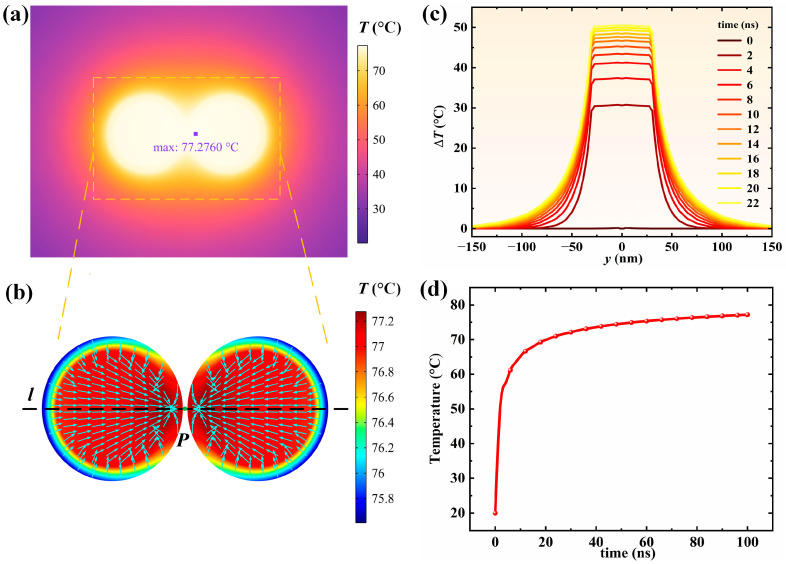
Thermal effect of the dimer. Wavelength *λ* = 502 nm, irradiance *I*_i_ = 1 × 10^9^ W/m^2^, the electric field oscillates along the *y*-axis and is incident along the positive *x*-axis onto the dimer with *R* = 12 nm, *t* = 3 nm, and *D* = 1 nm. At 100 ns; (**a**) heat transfer distribution of the dimer in water; (**b**) temperature distribution inside the particles, with arrows indicating the direction of the temperature gradient; (**c**) temperature distribution curves along the dimer axis (line *l*) at different times; (**d**) temperature variation at the particle center (point *P*) over time.

When the nanosphere dimer is exposed to light, the electrons inside absorb the energy of photons and move in the electric field, generating Joule heat. The magnitude of the heat generation power depends on the strength of the absorption performance of the nanospheres and the irradiance of the incident light. Through previous research, we have screened out the size of nanospheres with better absorption performance to study their photothermal properties. In the following research, we selected the core radius *R* of 12 nm, the shell thickness *t* of 3 nm, the particle spacing *D* of 1 nm, the SM refractive index *n*_m_ of 1.33, and the incident light with an irradiance of 1 × 10^9^ W/m^2^ at the resonance wavelength of 502 nm with a 0° incident angle. Figure 12 shows the photothermal characteristics of the Ag@TiO_2_ nanosphere dimer. After 100 ns of electromagnetic wave irradiation, the dimer’s temperature increase stabilizes, reaching a maximum of 77.3 °C. This temperature is suitable for water purification applications. From Figure 12c,d, it can be seen that the area with the most significant temperature increase is near the dimer, and the temperature increase decreases exponentially as the distance from the dimer increases. The temperature of the central particle of the dimer changes rapidly within 0 to 20 ns and gradually stabilizes after 20 ns. This suggests that nanosecond pulsed lasers can be used to efficiently excite the dimer to generate local high temperatures. Additionally, from Figure 12b,c, it can be seen that the highest temperature of the dimer is not at the particle gap but on the core–shell contact surface near the center of the dimer. The strong coupling effect of the dimer causes the hot electrons to concentrate on one side of the particle gap, but the presence of TiO_2_ blocks the heat dissipation of the Ag nanospheres, resulting in a decrease in the temperature at the center of the dimer gap. Fortunately, this decrease does not affect the overall temperature increase effect and can be ignored.

## 4. Conclusions

Regarding the application of Ag@TiO_2_ core–shell nanosphere dimer in photocatalytic water purification, this paper quantitatively investigates their optical absorption and scattering properties, local electric field enhancement, and photothermal effects using FEM and FDTD methods, and explores the formation mechanism of the multipole resonance mode of the dimer. The results show the following: (1) Decreasing the particle spacing leads to a redshift in the resonance wavelength, improved optical absorption and scattering, and stronger local electric field enhancement. (2) By changing the inner core radius and outer shell thickness of the particles, the resonance wavelength can be adjusted arbitrarily within the visible light range to meet application requirements. (3) As the SM refractive index increases, the resonance wavelength shifts towards the red and the local electric field enhancement weakens. (4) As the incident light azimuth angle increases, the resonance mode gradually changes from L-LSPR mode to T-LSPR mode. When the azimuth angle is 0°, the dimer exhibits optimal optical absorption and scattering performance. (5) After being irradiated with light for several tens of nanoseconds, the temperature of the dimer rapidly increases to a steady state, and the temperature at the core–shell interface near the dimer is the highest, while the temperature away from the dimer shows an exponential decay trend. Based on our discussion, we found that the higher the particle concentration (with a smaller spacing, approximately 2 nm), the thinner the shell (approximately 3 nm), and the particle with a core radius ranging from 12 to 20 nm has better catalytic performance for water purification. Smaller nuclear radius can result in a higher absorption performance, thereby generating a higher surface temperature promotes catalytic oxidation reactions in water and the elimination of bacteria and viruses. Larger core radius results in better scattering performance. The stronger scattering performance causes the electric field between the particles to be strongly enhanced, making the generation of hydroxyl and other active oxides more efficient and convenient. Quantum effects, which may be triggered when the particle spacing decreases to 1 nm, were not considered in this work but will be addressed in future improvements.

## Figures and Tables

**Table 1:** Relevant thermal parameters of Ag, TiO_2_, and H_2_O [36].

Material	Density (ρ [kg/m^3^])	Specific Heat Capacity (*C*_p_ [J/kg/K])	Thermal Conductivity (*k* [W/m/K])
Ag	10,500	235	429
TiO_2_	3800	686	6.7
H_2_O	997	4182	0.6

## Data Availability

The original contributions presented in this study are included in the article; further inquiries can be directed to the corresponding authors.

## References

[1] Garrick D.E., Hall J.W., Dobson A., Damania R., Grafton R.Q., Hope R., Hepburn C., Bark R., Boltz F., De Stefano L. (2017). Valuing Water for Sustainable Development. Science.

[2] Kümmerer K., Dionysiou D.D., Olsson O., Fatta-Kassinos D. (2018). A Path to Clean Water. Science.

[3] Mukherjee A., Coomar P., Sarkar S., Johannesson K.H., Fryar A.E., Schreiber M.E., Ahmed K.M., Alam M.A., Bhattacharya P., Bundschuh J. (2024). Arsenic and Other Geogenic Contaminants in Global Groundwater. Nat. Rev. Earth Environ..

[4] Münzel T., Hahad O., Lelieveld J., Aschner M., Nieuwenhuijsen M.J., Landrigan P.J., Daiber A. (2025). Soil and Water Pollution and Cardiovascular Disease. Nat. Rev. Cardiol..

[5] Li J., Zhang S., Guo L., Chen L., Yu Z. (2021). Chlorination Contributes to Multi-Antibiotic Resistance in a Pilot-Scale Water Distribution System. Water Supply.

[6] Beattie R.E., Skwor T., Hristova K.R. (2020). Survivor Microbial Populations in Post-Chlorinated Wastewater Are Strongly Associated with Untreated Hospital Sewage and Include Ceftazidime and Meropenem Resistant Populations. Sci. Total Environ..

[7] Yuan Q.-B., Guo M.-T., Yang J. (2015). Fate of Antibiotic Resistant Bacteria and Genes during Wastewater Chlorination: Implication for Antibiotic Resistance Control. PLoS ONE.

[8] Putra H.S.A., Ma’rufi I., Ellyke E. (2022). Analisis risiko kesehatan lingkungan sisa klor (Cl2) pada ZAMP perumda air minum tugu tirta kota malang. Pustaka Kesehat..

[9] Bolan N.S., Bell K., Krishan A.K., Chung J.-W. (2011). Irrigating Horticultural Crops with Recycled Water: An Australian Perspective. J. Hortic. Sci..

[10] Al-Mamun M.R., Kader S., Islam M.S., Khan M.Z.H. (2019). Photocatalytic Activity Improvement and Application of UV-TiO_2_ Photocatalysis in Textile Wastewater Treatment: A Review. J. Environ. Chem. Eng..

[11] Ouyang Z., Yang Y., Zhang C., Zhu S., Qin L., Wang W., He D., Zhou Y., Luo H., Qin F. (2021). Recent Advances in Photocatalytic Degradation of Plastics and Plastic-Derived Chemicals. J. Mater. Chem. A.

[12] Hashimoto K., Irie H., Fujishima A. (2005). TiO_2_ Photocatalysis: A Historical Overview and Future Prospects. Jpn. J. Appl. Phys..

[13] Park H., Park Y., Kim W., Choi W. (2013). Surface Modification of TiO2 Photocatalyst for Environmental Applications. J. Photochem. Photobiol. C.

[14] Lawless D., Serpone N., Meisel D. (1991). Role of Hydroxyl Radicals and Trapped Holes in Photocatalysis. A Pulse Radiolysis Study. J. Phys. Chem..

[15] Kesselman J.M., Weres O., Lewis N.S., Hoffmann M.R. (1997). Electrochemical Production of Hydroxyl Radical at Polycrystalline Nb-Doped TiO_2_ Electrodes and Estimation of the Partitioning between Hydroxyl Radical and Direct Hole Oxidation Pathways. J. Phys. Chem. B.

[16] Tada H., Jin Q., Kobayashi H. (2012). Prediction of the Main Route in the TiO_2_-photocatalyzed Degradation of Organic Compounds in Water by Density Functional Calculations. ChemPhysChem.

[17] Naya S., Kume T., Akashi R., Fujishima M., Tada H. (2018). Red-Light-Driven Water Splitting by Au(Core)–CdS(Shell) Half-Cut Nanoegg with Heteroepitaxial Junction. J. Am. Chem. Soc..

[18] Narayan M.R. (2011). Review: Dye Sensitized Solar Cells Based on Natural Photosensitizers. Renew. Sustain. Energy Rev..

[19] Garcia M.A. (2012). Surface Plasmons in Metallic Nanoparticles: Fundamentals and Applications. J. Phys. D Appl. Phys..

[20] Hong D., Lyu L.-M., Koga K., Shimoyama Y., Kon Y. (2019). Plasmonic ag@TiO_2_ Core–Shell Nanoparticles for Enhanced CO_2_ Photoconversion to CH_4_. ACS Sustain. Chem. Eng..

[21] Wumaier D., Tuersun P., Li S., Li Y., Wang M., Xu D. (2024). Light Absorption Analysis and Optimization of ag@TiO_2_ Core-Shell Nanospheroid and Nanorod. Nanomaterials.

[22] Liu Y., Lu X., Zhang R., Wang J., Zhou Z., Xia Y., Li N., Chen D., Zhou Z., Fan X. (2025). Local Polarization Piezoelectric Electric Field Promoted Water Dissociation for Hydroxyl Radical Generation under Ambient Humidity Condition. Adv. Mater..

[23] Li J., Cushing S.K., Bright J., Meng F., Senty T.R., Zheng P., Bristow A.D., Wu N. (2013). Ag@Cu_2_ O Core-Shell Nanoparticles as Visible-Light Plasmonic Photocatalysts. ACS Catal..

[24] Zhao J., Pinchuk A.O., McMahon J.M., Li S., Ausman L.K., Atkinson A.L., Schatz G.C. (2008). Methods for Describing the Electromagnetic Properties of Silver and Gold Nanoparticles. Acc. Chem. Res..

[25] Myroshnychenko V., Rodríguez-Fernández J., Pastoriza-Santos I., Funston A.M., Novo C., Mulvaney P., Liz-Marzán L.M., García De Abajo F.J. (2008). Modelling the Optical Response of Gold Nanoparticles. Chem. Soc. Rev..

[26] Amirjani A., Sadrnezhaad S.K. (2021). Computational Electromagnetics in Plasmonic Nanostructures. J. Mater. Chem. C.

[27] Hövel H., Fritz S., Hilger A., Kreibig U., Vollmer M. (1993). Width of Cluster Plasmon Resonances: Bulk Dielectric Functions and Chemical Interface Damping. Phys. Rev. B.

[28] Kreibig U., Vollmer M. (1995). Optical Properties of Metal Clusters.

[29] Johnson P.B., Christy R.W. (1972). Optical Constants of the Noble Metals. Phys. Rev. B.

[30] Bohren C.F., Huffman D.R. (1983). Absorption and Scattering of Light by Small Particles.

[31] Moroz A. (2011). Electron Mean-Free Path in Metal-Coated Nanowires. J. Opt. Soc. Am. B.

[32] Sharma R., Sarkar A., Jha R., Kumar Sharma A., Sharma D. (2020). Sol-gel–Mediated Synthesis of TiO_2_ Nanocrystals: Structural, Optical, and Electrochemical Properties. Int. J. Appl. Ceram. Technol..

[33] Alaee R., Rockstuhl C., Fernandez-Corbaton I. (2018). An Electromagnetic Multipole Expansion beyond the Long-Wavelength Approximation. Opt. Commun..

[34] Schwinger J., DeRaad L.L., Milton K.A., Tsai W.-Y. (1998). Classical Electrodynamics.

[35] Baffou G., Quidant R., García De Abajo F.J. (2010). Nanoscale Control of Optical Heating in Complex Plasmonic Systems. ACS Nano.

[36] Haynes W.M. (2016). CRC Handbook of Chemistry and Physics.

[37] Brandl D.W., Oubre C., Nordlander P. (2005). Plasmon Hybridization in Nanoshell Dimers. J. Chem. Phys..

